# Anesthetic Management for a Child With a Newly Identified Mitochondrial Disease SLC25A46 Mutation: A Case Report

**DOI:** 10.7759/cureus.47076

**Published:** 2023-10-15

**Authors:** Thong Nguyen, Sarah Shabot, David Yngve, Amr Abouleish

**Affiliations:** 1 Anesthesiology and Perioperative Medicine, Texas Children's Hospital, Houston, USA; 2 Anesthesiology and Perioperative Medicine, University of Texas Medical Branch, Galveston, USA; 3 Orthopedic Surgery and Rehabilitation, University of Texas Medical Branch, Galveston, USA

**Keywords:** femoral osteotomy, intra-iliac osteotomy, anesthetic management, cerebral palsy, mitochondrial disease, slc25a46 mutation

## Abstract

SLC25A46 mutation is a newly recognized mitochondrial mutation causing neurological and muscular abnormalities. We describe a first-ever report of the anesthetic management of a seven-year-old boy with an SLC25A46 mutation during a major orthopedic procedure. The patient was nonverbal and presented with cerebral visual impairment, torticollis, and lower extremity contractures. Because of his new diagnosis of mitochondrial disease and history of delayed awakening after anesthesia, we performed general anesthesia with sevoflurane, a low-dose ketamine infusion, and small doses of fentanyl while avoiding propofol and maintaining normoglycemia and normothermia. No postoperative complications were noted during the recovery period.

## Introduction

Mitochondria are organelles that are essential for aerobic metabolism and energy production. Recently, researchers have discovered mutations in SLC25A46, a mitochondrial outer membrane protein that causes a wide disorder spectrum, including multiple neuropathies, optic atrophy, progressive myoclonic ataxia, and lethal congenital pontocerebellar hypoplasia [1-3]. While there is literature about the role of SLC25A46 in mitochondria and pathophysiology in cell and mouse models, none has referenced the anesthetic management of patients with this specific mutation. Since the mitochondrial disorder phenotype is heterogeneous, case reports continue to be valuable.

Mitochondrial diseases (MD) create multiple challenges, as any catabolic stress, such as fasting or surgery, can rapidly lead to acidosis and hypoglycemia. The severity of co-morbidities associated with the mitochondrial disorder requires that an anesthetic plan be individualized based on each patient's disease. Finally, the anesthetic agents must be carefully selected to avoid triggering metabolic decompensation, rhabdomyolysis, and profound hyperkalemia. 

We describe the successful anesthetic management of a child diagnosed with compound heterozygous mutations in the SLC25A46 gene undergoing a major orthopedic surgery to reduce bilateral hip subluxation, contractures, and spasticity. Written Health Insurance Portability and Accountability Act (HIPAA) authorization was obtained from the patient’s parents for the publication of this case report. The genetic sequencing that supports the patient's diagnosis cannot be provided due to the risk of violating HIPPA-protected patient information and privacy.

## Case presentation

The patient is a seven-year-old male who had early speech and motor delay. By 13-20 months of age, he was hospitalized monthly for sudden head dropping, recurrent vomiting, irritability, and nocturnal myotonic jerking of lower extremities. By 25 months of age, he had a catastrophic febrile seizure, resulting in persistent difficulty sitting and standing. The patient developed global developmental delay, optic nerve atrophy, and cerebral palsy with torticollis. He was eventually diagnosed with compound heterozygous variants in the SLC25A46 gene. 

**Figure 1:**
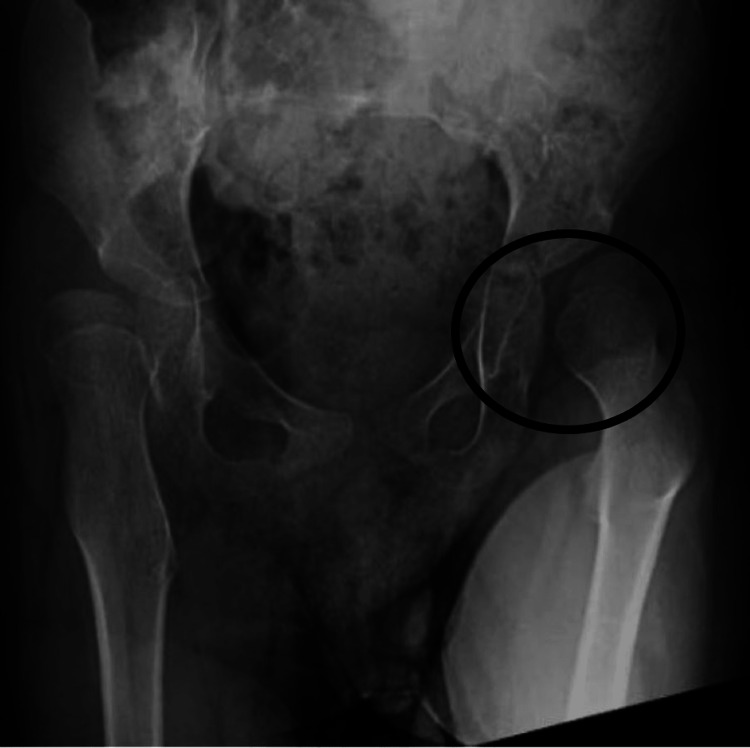
Pre-surgery X-ray showing significant left hip subluxation and malrotation of the femoral head.

**Figure 2:**
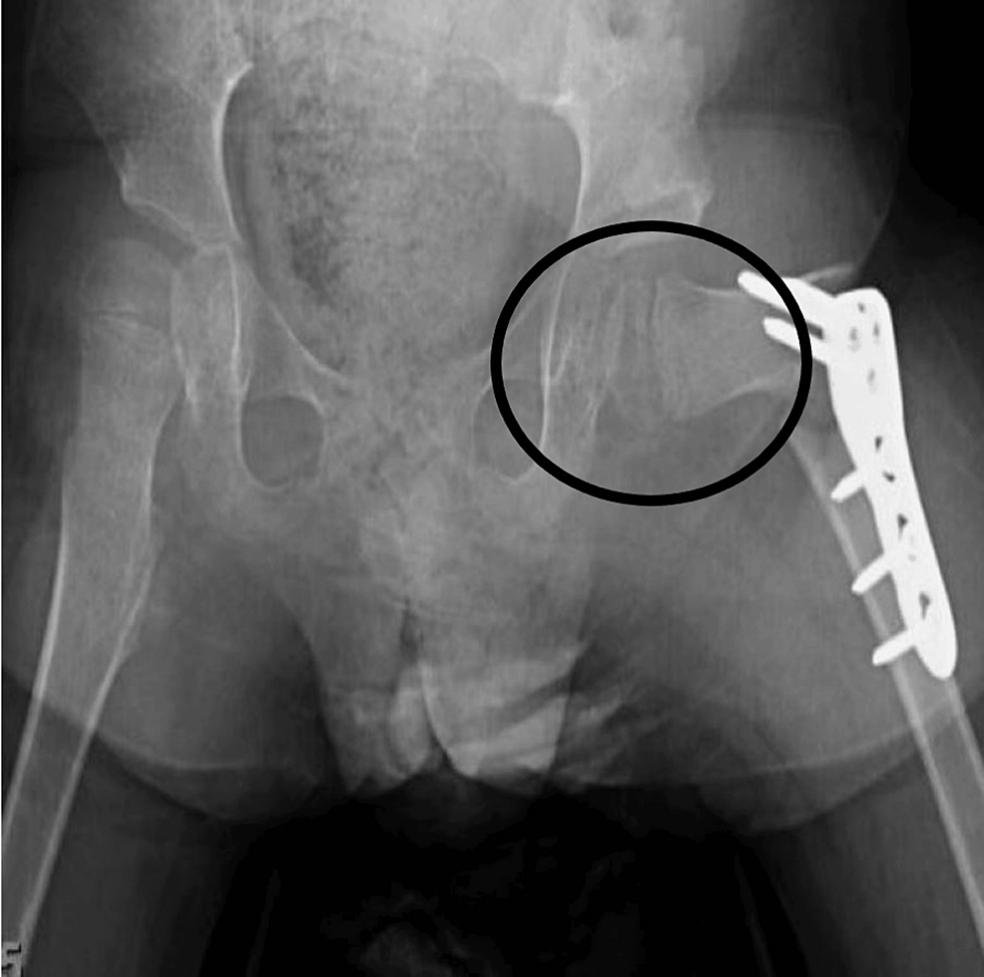
Post-surgery X-ray showing reconstruction of the left femoral head and hardware to allow for mobility and rotation.

Due to worsening hip subluxation, contractures, and spasticity, he presented for a left intra-iliac osteotomy with open reduction and femoral osteotomy along with selective percutaneous myofascial lengthening of bilateral hip adductors and gastrocnemius. The alcohol neurolysis of the obturator, iliocostalis dorsi, and iliocostalis lumborum nerves was performed to relieve spasticity. For post-operative pain control, the surgeon implanted catheters at the surgical site for local anesthesia infusion via a portable dispenser. The pre- and post-surgery hip X-rays are included in Figures 1, 2. 

Pre-anesthesia evaluation revealed that he was 15.7 kg and nonverbal with torticollis, lower extremity contractures, muscle weakness, and was wheelchair-bound. His parents reported a previous delayed emergence for more than six hours after a magnetic resonance imaging scan. The anesthesia report for this scan included 1.0 minimum alveolar concentration (MAC) of sevoflurane and 0.5 mcg/kg of intravenous dexmedetomidine. Because the patient is already at risk of respiratory complications based on his condition, we want to avoid long-acting opioids to prevent respiratory depression. The anesthetic plan also includes multi-modal analgesia and avoids propofol because of its effect on mitochondrial electron transfer pathways. 

The patient received oral midazolam (0.5 mg/kg) and acetaminophen (10 mg/kg) as premedication. After the standard monitors were placed in the operating room, the patient underwent inhalational induction with 8% sevoflurane, followed by the placement of an intravenous catheter and oral endotracheal intubation after vocal cord topicalization. A 22-gauge right radial arterial line was placed for hemodynamic monitoring and frequent blood gases. Anesthesia was maintained with 0.7 MAC of sevoflurane and a ketamine infusion (0.3 mg/kg/hour). After the surgeon finished with the alcohol neurolysis portion, rocuronium was given to facilitate surgery with a 20 mg initial dose and then 5 mg every hour. Intermittent boluses of intravenous fentanyl (1 mcg/kg) were administered. Intravenous dexamethasone (0.15 mg/kg) and ondansetron (0.15 mg/kg) were used to prevent postoperative nausea and vomiting. To minimize blood loss, the patient received a loading dose of 50 mg/kg intravenous tranexamic acid over 30 minutes, followed by an infusion of 5 mg/kg/hour until surgical closure. The 5% dextrose in 0.9 normal saline was infused at a rate of 52 ml/hour as a maintenance fluid. 

During surgery, the patient maintained a mean arterial pressure between 60 and 70 mmHg, heart rates in the range of 80-90s, a 98% oxygen saturation, and a body temperature of 37°C. The surgery lasted seven hours. No intraoperative complications occurred. The estimated blood loss was 100 ml. The patient received intravenous acetaminophen (10 mg/kg), ketorolac (0.5 mg/kg), and 4 mg/kg of sugammadex for reversal. Upon confirmation of a 100% 4/4 train-of-four, the patient was extubated in the operating room and transferred to the post-anesthesia care unit (PACU). Once stable for two hours, he was admitted to the pediatric intensive care unit as part of the policy for monitoring. The patient had an unremarkable postoperative course and was discharged on postoperative day five.

## Discussion

There is a wide spectrum of clinical features associated with SLC25A46 mutations. In the literature, there are about 10 patients with SLC25A46 variants, some of which are lethal, resulting in early death in three patients (four weeks, three months, and 13 months of age). All patients reported in the literature share optic atrophy and axonal neuropathy, including ours. In vitro studies showed that SLC25A46 mutations cause abnormal mitochondrial morphology and dysfunction, which lead to energy deficiencies in distal axons and synaptic terminals, resulting in neurodegeneration and demyelination [4]. The SLC25A46 -/- mice demonstrated a reduction in respiratory complex activity and adenosine triphosphate (ATP) production, suggesting cellular metabolic dysfunction [4]. The exact molecular mechanism of SLC25A46 function remains elusive, as it can play a role in mitochondrial fusion and fission dynamics and the maintenance of mitochondrial cristae [5].

How do anesthetic agents affect mitochondrial function?

The selection of general anesthetics has remained an ongoing discussion topic. Some studies showed that volatile anesthetics depress oxidative phosphorylation in isolated mitochondria, especially complex I [6,7]. In their study of guinea pig myocytes, Hanley et al. estimated that inhalational agents at >2 MAC could inhibit the electron transport chain by 20-25% [8]. However, clinically, Driessen et al. and Muravchick et al. showed no significant adverse effects of inhalational anesthetics [9,10]. 

Propofol in patients with mitochondrial disease (MD), especially children, has been reported to lead to the development of metabolic acidosis similar to propofol infusion syndrome (PRIS). Propofol has been described to impair mitochondrial oxidative phosphorylation at the coenzyme Q site [11]. Propofol also disrupts free fatty acid utilization by uncoupling β-oxidation and inhibiting carnitine palmitoyl transferase I. Although PRIS is more associated with high-dose prolonged propofol infusion, there was a case report of metabolic encephalopathy, lactic acidosis, and stroke-like syndrome exacerbation after a single dose of propofol in a child with mitochondrial disease [12]. Hence, many experts recommend avoiding propofol in children with mitochondrial diseases. 

In an in vitro study of the neurotoxicity of ketamine to human-induced pluripotent stem cell-derived neurons, Ito et al. found that ketamine reduced ATP production and promoted mitochondrial fission due to the inhibition of respiratory complexes I and V. This occurred with ketamine at 100 𝜇M plasma level [13]. However, this plasma level is highly unlikely to encounter with typical anesthetic dosing of ketamine. Fan et al. showed that at sub-anesthetic dosing, ketamine prevented neuronal apoptosis by elevating autophagy markers and reducing mTOR activation in treated Parkinson's disease (PD) mice as compared to untreated PD mice [14]. 

What does our anesthesia protocol include?

Since these patients are often vulnerable to metabolic decompensation at baseline, it is crucial to minimize dehydration and avoid prolonged fasting. While adherent to the eight-hour NPO (nil per os) for solid food, the patient was encouraged to have clear liquid up to one hour prior to the surgery. The 5% dextrose in 0.9 normal saline infusion and the upper body forced-air warming blanket were used to maintain normoglycemia and normothermia.

Propofol is avoided based on its effects on mitochondrial metabolism, and sevoflurane at 0.7 MAC is unlikely to affect patients clinically. Succinylcholine is avoided due to the risk of hyperkalemia and rhabdomyolysis in mitochondrial disease patients with underlying muscle wasting. Non-depolarizing muscle blockade (rocuronium) is judiciously titrated, and sugammadex is used for reversal. 

The subanesthetic ketamine infusion provided excellent analgesia and neuroprotection while minimizing the opioid and MAC requirements of volatile anesthetics. At this dosing, ketamine has a minimal physiologic impact on mitochondria, but it is associated with a low incidence of mild psychomimetic symptoms [15]. Hence, it was turned off after four hours of infusion to minimize the side effects of ketamine and prepare for the emergence. 

Central neuraxial blockades are not an option in this case due to the need of stimulating and localizing the focal nerves for alcohol neurolysis. The single-shot ultrasound-guided lumbar plexus block can be an alternative or supplement to our anesthetic plan, but care should be taken to avoid inadvertent intravascular injection and retroperitoneal bleeding.

## Conclusions

The SLC25A46 mutation is a rare, newly recognized mitochondrial mutation that requires ongoing research. Our patient also had mixed-tone cerebral palsy and muscle weakness, which is an atypical presentation of mitochondrial diseases. We demonstrated the safe and effective use of multimodal analgesia while minimizing long-acting opioids and avoiding triggers for metabolic decompensation such as propofol. The limitation in our report is the lack of exploration or inclusion of a peripheral nerve blockade. However, we made a conservative decision not to consider this option because we were uncertain about how local anesthetics can affect neurons and the conduction pathway in SLC25A46 mutation disorders. Finally, there is still a knowledge gap regarding how each anesthetic agent affects this mutation and mitochondrial function specifically.
